# Investigation of the Insulation Characteristics of TPU/EP Composites Under Cold Thermal Shock

**DOI:** 10.3390/ma18081840

**Published:** 2025-04-17

**Authors:** Guoqing Yang, Nan Ding, Chaolu Jiang, Peizhi Yang, Qingqing Gao, Yichen He, Lu Han

**Affiliations:** School of Electrical Engineering, Xi’an University of Technology, Xi’an 710054, China; victoriajcl@163.com (C.J.); 17789285095@163.com (P.Y.); 18789460762gqq0310@xaut.edu.cn (Q.G.); 19909230189@163.com (Y.H.); hl25002x@163.com (L.H.)

**Keywords:** EP, thermoplastic polyurethane, thermal shock, dielectric properties, mechanical properties

## Abstract

To improve the issue of the decreased toughness and electrical performance of epoxy resin (EP) in thermal shock environments, we prepared thermoplastic polyurethane elastomer (TPU)-filled modified EP composites. We also studied the mechanical and electrical performance of these composites, which had different TPU filling contents, under thermal shock conditions. The results indicated that after 240 h of thermal cycling between −15 °C and 100 °C, the TPU/epoxy composites, when compared to unmodified EP, exhibited a 10.1% enhancement in their elastic modulus, a 15.3% increase in their elongation at break, a 22.3% improvement in their tensile strength, and a 47.8% increase in their impact strength. Moreover, their volume resistivity increased by 10.5% and their AC breakdown strength improved by 52.1%. In contrast, their dielectric constant and dielectric loss experienced reductions of 40.2% and 7.5%, respectively. This study demonstrates that introducing flexible TPU molecular chains into the resin significantly enhances the toughness of EP structures. Additionally, the new cross-linked structures formed within the TPU/EP composites improve their insulation performance under thermal shock conditions.

## 1. Introduction

Epoxy resin (EP) is one of the primary solid insulating materials used for electrical equipment due to its superior insulation properties. However, the three-dimensional network structure of EP causes it to become brittle and hard in low-temperature environments. This brittleness increases its susceptibility to cracking and damage. In high-temperature conditions, internal stresses within EP can result in cracking and delamination, adversely affecting its mechanical and electrical properties [1]. Therefore, researchers have emphasized the need to enhance the toughness of EP while maintaining its inherent strength and electrical characteristics. This enhancement is crucial for its adapting to variations in complex environments.

Common toughening methods for EP include the introduction of rubber, thermoplastic resins, inorganic nanoparticles, hyperbranched polyesters, block copolymers, liquid crystal elastomers, bio-based materials, and core–shell polymers [2]. The addition of rubber significantly enhances the material’s toughness [3]; however, it causes a drastic reduction in its glass transition temperature. The addition of inorganic nanoparticles and core–shell polymers can improve the toughness and strength of the material. However, both of them have significant agglomeration problems, which result in poor filling effects [4]. Hyperbranched polyesters and liquid crystal elastomers provide excellent toughening effects, yet their high costs and susceptibility to aging and performance degradation at elevated temperatures present challenges [5]. Block copolymers enhance the toughness and mechanical properties of composites; however, compatibility issues with EP have arisen. The bio-based toughening of EP offers good environmental benefits, yet these materials can show performance instability due to environmental influences. Incorporating thermoplastic resins into EP enhances its toughness while improving its mechanical strength, with good material compatibility.

Polyurethane (PU), as a thermoplastic resin, effectively cross-links with EP, leading to a more extensive secondary network within the composites. This structure reduces EP’s brittle fracture in low-temperature environments and enhances its thermal expansion performance at high temperatures, while preventing changes in the other properties of the composites [5]. TPU is preferred for adding to EP due to its excellent elasticity. However, the performance of the resulting composites under extreme cold and heat conditions warrants further investigation.

Therefore, this study introduces thermoplastic polyurethane elastomer (TPU) into the EP formulation system and conducts experiments under thermal shock conditions. The focus is on examining the impact of TPU on the mechanical and insulation properties of EP composites, providing a theoretical basis for the engineering application of TPU/EP composites.

## 2. Material Preparation and Experimental Methods

### 2.1. Raw Materials and Manufacturers

Table 1 shows the raw materials selected for the experiment and their manufacturers. All raw materials were over 99% pure.

### 2.2. Material Preparation

**Figure 1:**
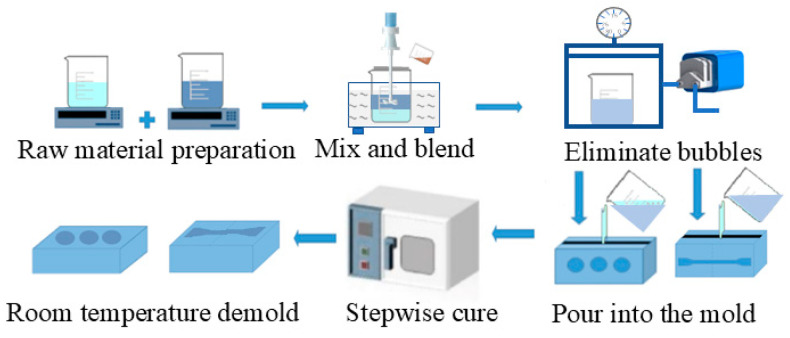
Sample preparation process for TPU/EP composite materials.

The preparation process for the TPU/EP composites is illustrated in Figure 1. TPU was placed in a beaker and DMF was added at a TPU-to-DMF mass ratio of 2:5. The mixture was subjected to mechanical ultrasonic stirring at 50 °C for 120 min, with an ultrasonic frequency of 20 kHz and a power of 200 W. After stirring, considering the potential impact of solvent residues on the composite’s performance, the TPU solution was vacuum-dried at 50 °C for 24 h to ensure complete DMF evaporation and eliminate its influence on the composite system.

EP was preheated in an 80 °C oven for 10 min to reduce its viscosity. The processed TPU solution was then gradually dripped into the EP matrix at mass fractions of 0 wt%, 10 wt%, 12.5 wt%, 16.7 wt%, 25 wt%, and 50 wt% (TPU-to-EP mass ratios of 0:1, 1:9, 1:7, 1:5, 1:3, and 1:1, respectively). During this process, low-speed stirring at 300 rpm for 10 min was applied to minimize the effects of shear force on the system’s uniformity, followed by an increase in the stirring speed to 800 rpm for 10 min to further enhance TPU dispersion in the EP matrix.

Subsequently, a curing agent, toughening agent, and accelerator were introduced at a mass ratio of 100:80:20:3 (EP–curing agent–toughening agent–accelerator). The mixture was then subjected to mechanical ultrasonic stirring for 30 min to obtain an uncured composite. After vacuum degassing at 60 °C, the composite mixture was poured into a polytetrafluoroethylene mold and subjected to gradient curing at 100 °C for 1 h, 110 °C for 1 h, 120 °C for 1 h, 140 °C for 1 h, and 150 °C for 1 h. Finally, the cured composite was allowed to cool to room temperature before being demolded and collected for further testing.

### 2.3. Testing Methods

Mechanical and electrical performance tests were conducted on composite materials with different filler contents. The tests were performed at room temperature (25 °C), with each experiment repeated five times. After obtaining 15 data points, the average value was calculated to avoid experimental errors. The thermal shock cycling test involves subjecting the composites to cycles between 100 °C and −15 °C for 240 h, with a cycle occurring every 12 h. Every 48 h, samples were extracted for testing at room temperature. The processed data are presented with the timeline on the *x*-axis, with 0 h being before the thermal shock, and the specific times corresponding to the performance of the composite after different thermal shock durations.

The electrical performance test specimens were circular thin disks with a diameter of 50 mm and a thickness of 1 mm. Volume resistivity was measured using the volt–ampere method, employing the UC750S impedance meter manufactured by U-CHEER Electronics, China. A DC voltage of 3000 V was applied, and the measurement duration was set to 60 s. The relative permittivity and dielectric loss tangent were evaluated using the GWS-4 high-voltage dielectric loss tester produced by Wuhan Kexin Electric Power Equipment Co., Ltd., China. The local discharge inception voltage and short-time AC breakdown field strength of the composite materials were determined using a YDQ15 tester, also manufactured by Wuhan Kexin Electric Power Equipment Co., Ltd., China, with a 20 mm diameter stainless steel spherical electrode pair and a gradient voltage ramp-up rate of 500 V/s. The material’s infrared absorption was tested in the wavelength range of 4000 to 500 cm^−1^ using the IRTracer100 spectrometer produced by Shimadzu Corporation, Japan, with its testing mode set to Attenuated Total Reflectance (ATR) and 32 scans performed. The microstructure of the samples was observed using the GeminiSEM360 field-emission scanning electron microscope from Zeiss, Oberkochen, Germany. Differential scanning calorimetry (DSC) was performed using the DZ-DSC300 differential scanning calorimeter, produced by Nanjing Dazhan Testing Instruments Co., Ltd., China, under a nitrogen atmosphere, with a heating rate of 10 °C/min and a temperature range of 25 °C to 250 °C.

## 3. Test Results

### 3.1. Main Raw Materials

**Figure 2:**
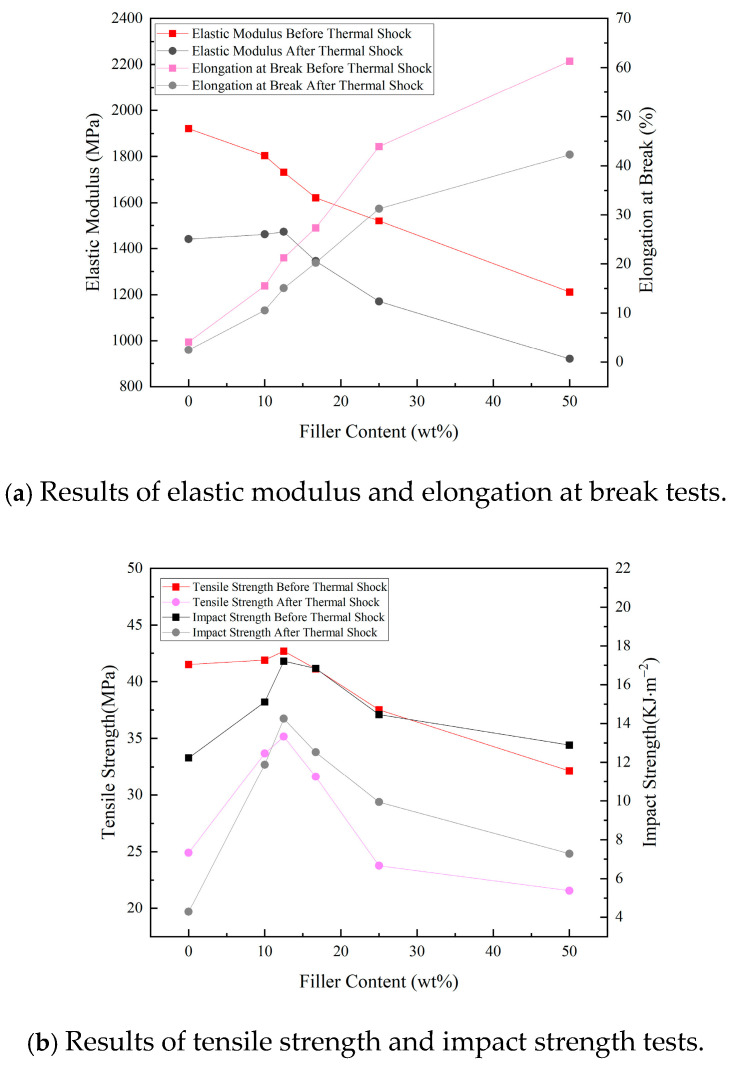
Results of mechanical property testing.

Figure 2 shows the test results of the elastic modulus, tensile strength, elongation at break, and impact strength of the composites. The data in Figure 2a indicate that after thermal shock, the elastic modulus of the EP without TPU filler decreased by 480.25 MPa. The composites with TPU filler contents of 10–16.7 wt% showed a maximum decrease in their elastic modulus of 342.76 MPa, while those with 25–50 wt% filler showed decreases between 290.64 MPa and 249.6 MPa. The elongation at the break of the EP without TPU filler decreased by 40.1%. In contrast, the composites with TPU filler contents of 10–16.7 wt% exhibited a maximum decrease of 32%, and those with 25–50 wt% filler showed decreases ranging from 28.7% to 31.1%.

The data in Figure 2b reveal that after thermal shock, the tensile strength of the EP without TPU filler decreased by 16.6 MPa. The composites with TPU filler contents of 10–16.7 wt% showed a maximum decrease in tensile strength of 9.51 MPa, and those with 25–50 wt% filler experienced decreases between 10.57 MPa and 13.76 MPa. The impact strength of the EP without TPU filler dropped by 7.96 kJ·m⁻^2^. In comparison, the composites with TPU filler contents of 10–16.7 wt% exhibited a maximum decrease of 4.32 kJ·m⁻^2^, and those with 25–50 wt% filler showed decreases between 4.52 kJ·m⁻^2^ and 5.61 kJ·m⁻^2^.

The experimental results demonstrated that as the TPU filler content increased, the elastic modulus of the composites decreased, their elongation at break increased, and both their tensile strength and impact strength initially increased and then decreased. After the thermal shock, the composites containing 12.5 wt% TPU filler exhibited the slightest reduction in elastic modulus, with a decrease of only 15%, as well as the smallest decrease in tensile strength, at only 17.6%, and the smallest decrease in impact strength, at only 17.2%. The composite containing 16.7 wt% TPU filler showed the smallest decrease in elongation at break, with a reduction of only 26.2%.

### 3.2. Electrical Performance Test Results

#### 3.2.1. Volume Resistivity

**Figure 3:**
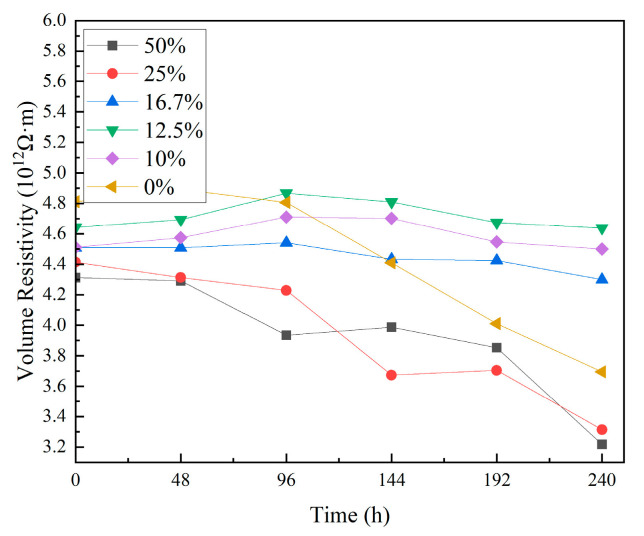
Results of volume resistivity test.

Figure 3 shows the test results of the composites’ volume resistivity before and after thermal shock. The data indicate that the volume resistivity of the composites decreases as the duration of the thermal shock test increases. Specifically, for the unfilled EP, its volume resistivity decreased by $1.12\times 10^{12}\;\Omega \cdot m$. In composites with TPU contents ranging from 10 wt% to 16.7 wt%, the volume resistivity decreased by up to $0.21\times 10^{12}\;\Omega \cdot m$. For composites with 25 wt% to 50 wt% TPU, their volume resistivity decreased within the range of $1.05\times 10^{12}\;\Omega \cdot m$ to $1.15\times 10^{12}\;\Omega \cdot m$.

These results demonstrate that the unfilled EP exhibits the most significant decrease in volume resistivity, reaching 23.3%. The composites with 10 wt% to 16.7 wt% TPU show the slightest change in volume resistivity, with reductions ranging from 0.2% to 4.7%.

#### 3.2.2. Relative Permittivity

**Figure 4:**
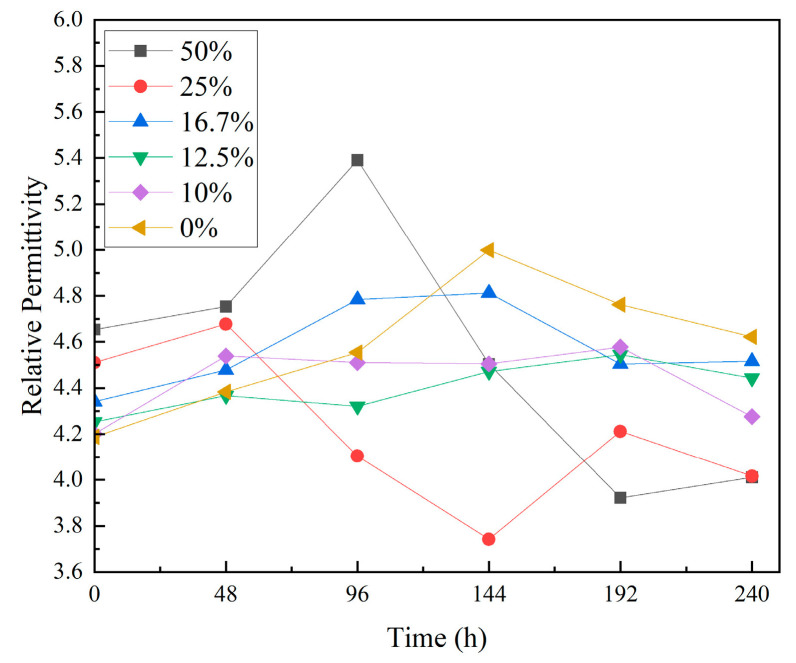
Results of relative permittivity test.

Figure 4 shows the relative permittivity test results before and after thermal shock. The data indicate that as the thermal shock time increases, the relative permittivity of the composites first rises and then falls. After 240 h of thermal shock, the relative permittivity of the unfilled EP increased by 43.4%. The maximum increase in composites with 10 wt% to 16.7 wt% TPU was 17.5%. For composites with 25 wt% to 50 wt% TPU, the relative permittivity decreased by 49.5% to 64.3%.

These results show that thermal shock causes the unfilled EP to significantly increase its relative permittivity. Composites with 10 wt% to 16.7 wt% TPU show a slight increase in relative permittivity, while composites with 25 wt% to 50 wt% TPU show an apparent decrease.

#### 3.2.3. Dielectric Loss

**Figure 5:**
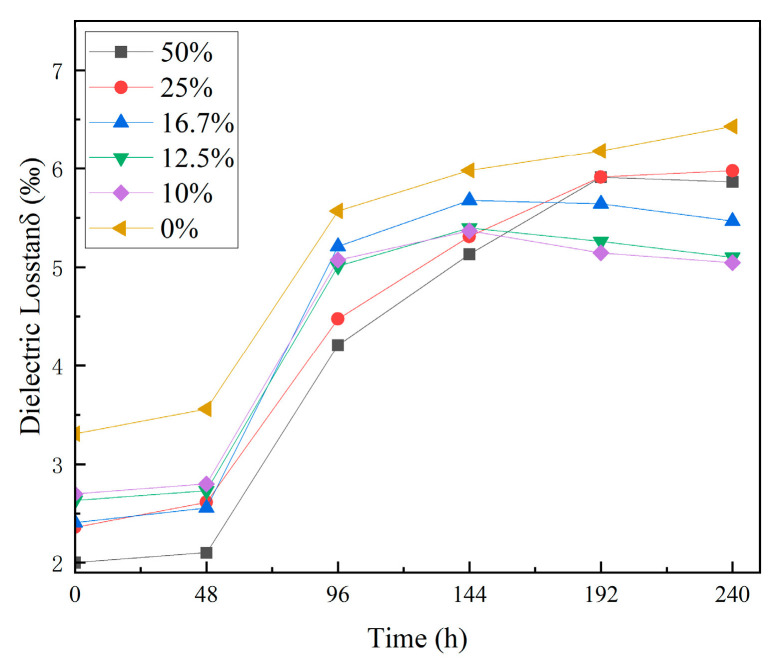
Results of dielectric loss test.

Figure 5 shows the dielectric loss test results for the composites before and after thermal shock. The data indicate that after 48 h of thermal shock, the dielectric loss of the composites slightly increased. From 48 h to 240 h, the dielectric loss rose more significantly. After 240 h of thermal shock, the dielectric loss of unfilled EP increased by 94.3%. For composites with 10 wt% to 16.7 wt% TPU, the increase ranges from 86.8% to 93.5%. For composites with 25 wt% to 50 wt% TPU, the increase is between 153.1% and 193.3%.

The results in Figure 5 suggest that unfilled EP experiences a significant increase in its dielectric loss. Adding TPU reduces the rate of the increase in dielectric loss for EP composites. Composites with 10 wt% to 16.7 wt% TPU exhibit lower dielectric losses than other concentrations.

#### 3.2.4. Partial Discharge Inception Voltage

**Figure 6:**
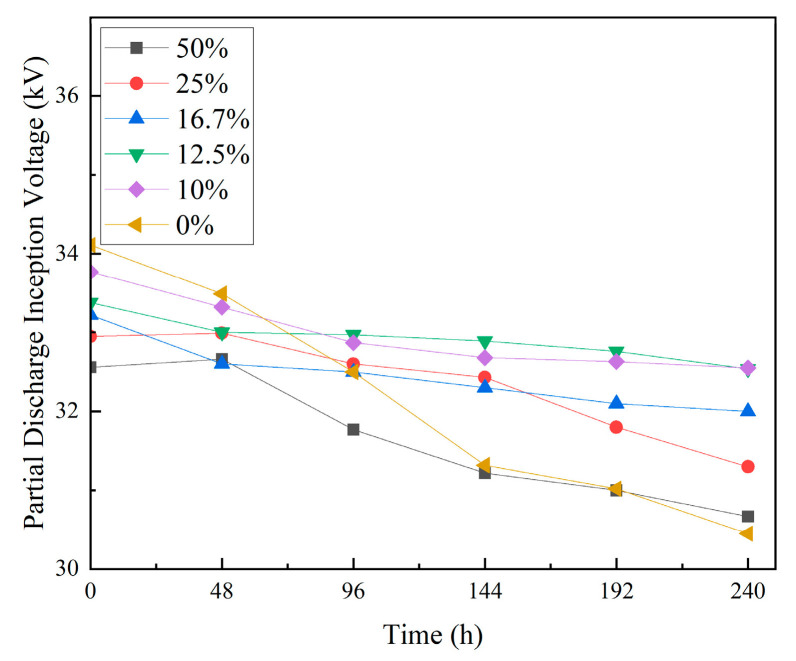
Results of PDIV.

Figure 6 presents the test results for the partial discharge inception voltage (PDIV) of the composites before and after thermal shock. As shown, with an increasing thermal shock duration, the PDIV of the composites gradually decreases. After 240 h of thermal shock, the unfilled EP experienced the most significant drop in PDIV, decreasing by 3.66 kV compared to the start of the experiment. For composites with 10 wt% to 16.7 wt% TPU, the PDIV decreased by a maximum of 1.22 kV, while for composites with 25 wt% to 50 wt% TPU, the PDIV decreased within the range of 1.65 kV to 1.89 kV.

These results suggest that adding TPU improves the PDIV of EP composites after thermal shock. Among the different TPU content levels, composites with 10 wt% to 16.7 wt% show a relatively higher PDIV, approximately 2.5% to 3.7% higher than that of unfilled EP.

#### 3.2.5. Short-Term AC Breakdown Field Strength

**Figure 7:**
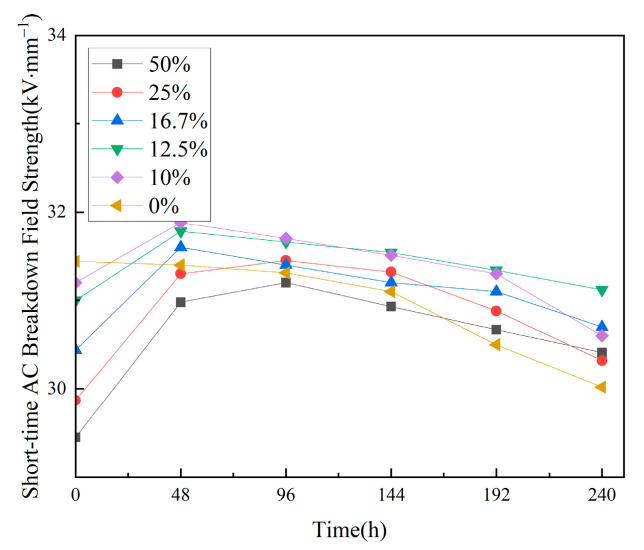
Results of AC breakdown strength test.

Figure 7 presents the test results for the short-term AC breakdown strength of the composites before and after thermal shock. The data show that before the thermal shock, the AC breakdown strength of TPU-filled EP composites ranges from 30.4 kV·mm^−1^ to 31.2 kV·mm^−1^, which is slightly lower than that of unfilled EP. After thermal shock, the breakdown strength of the unfilled EP drops quickly. In contrast, TPU-filled epoxy composites show different levels of improvement. After 192 h of thermal shock, the breakdown strength of all TPU/EP composites exceeds that of the unfilled epoxy resin.

These results indicate that TPU slightly reduces the AC breakdown strength of EP composites. However, thermal shock significantly slows down the decrease in their breakdown strength. The results also show that unfilled EP’s short-term AC breakdown strength decreases the most, by 4.5%. Composites with 10 wt% to 12.5 wt% TPU show a 0.4% to 1.9% reduction, while those with 16.7 wt% to 50 wt% TPU experience an increase of 1.5% to 3.2%.

### 3.3. Microscopic Morphology Test Results

#### 3.3.1. Infrared Spectral Test Results

**Figure 8:**
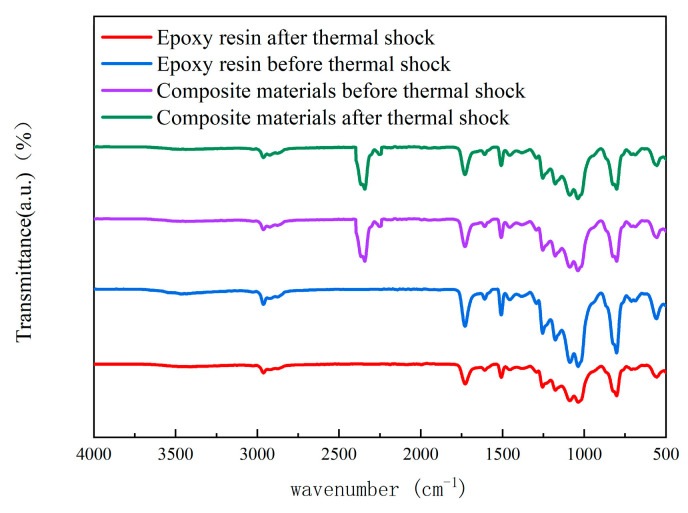
Infrared spectra of the composite material.

The composite material with a filler content of 12.5 wt% was selected for infrared spectroscopy testing. Figure 8 presents the infrared spectrum of this composite. Figure 8 shows a hydroxyl–OH absorption peak at 3500 cm^−1^. The stretching vibration peak of N—H is found in the 3300 to 3500 cm^−1^ range. The C—H stretching vibration peak in the methyl l group is observed from 3000 to 2750 cm^−1^. A stretching vibration peak of N=C=O appears between 2200 and 2250 cm^−1^. The characteristic peak for esters is at 1725 cm^−1^.

The absorption peak at 1680 cm^−1^ corresponds to the C=O bond in carbamate. The absorption peak at 1500 cm^−1^ is attributed to the phenyl ring. The C—N stretching vibration peak in phenyl carbamate appears at 1350 cm^−1^. Bending vibration peaks of C—H are present at 1340 to 1470 cm^−1^ and at 1000 cm^−1^. An absorption peak for epoxy is found at 1251 cm^−1^. The characteristic peak for epoxy appears at 900 cm^−1^. These peaks indicate that TPU and EP have partially cross-linked to form carbamate.

In Figure 8, it can be seen that the peak values of the isocyanate groups in TPU and the characteristic peaks in the EP decrease after thermal shock. This indicates an increase in the secondary curing cross-linked structure. The ester peaks also decrease, suggesting that the carbamate is decomposed to produce alkyl groups. The decrease in the alkyl peak values indicates further oxidation. The bending vibration peaks of O—H in the range of 1410 to 1440 cm^−1^ and the C—O stretching peaks from 1000 to 1300 cm^−1^ persist, indicating the formation of aldehyde and carboxylic acid groups.

#### 3.3.2. DSC Test Results

**Figure 9:**
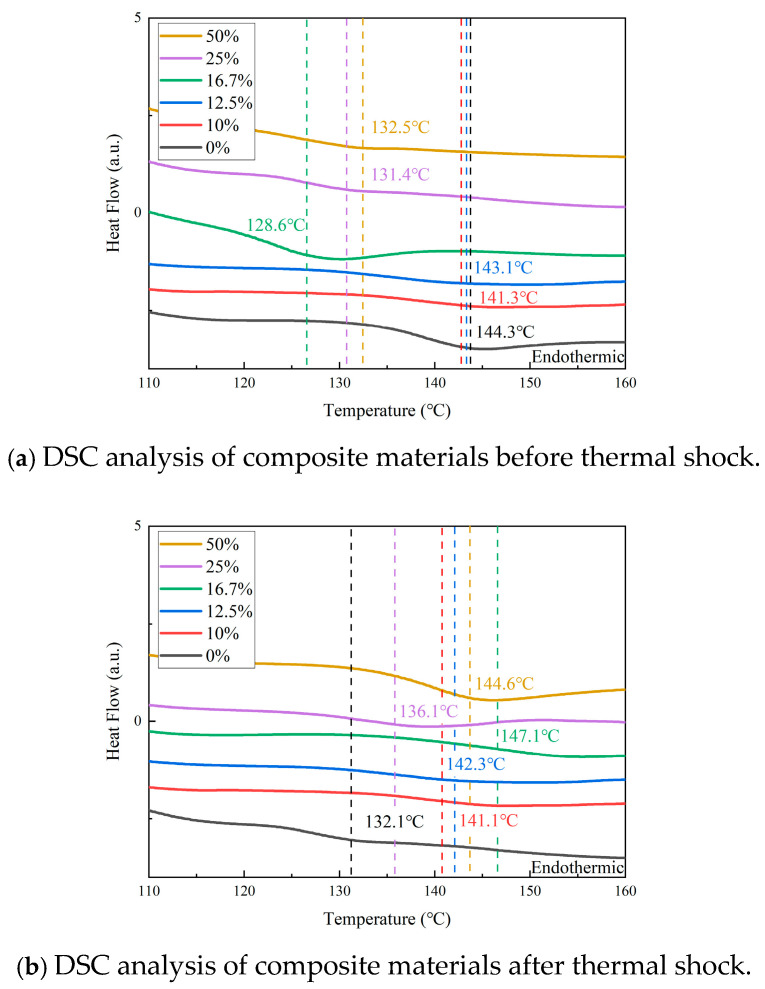
DSC, differential scanning calorimetry, of composite materials.

Figure 9 shows the DSC test results for the composite materials before and after thermal shock. Figure 9a shows that the glass transition temperature (Tg) of the TPU-filled epoxy (EP) composites ranges from 128.6 to 143.1 °C, which is lower than that of pure EP. After the thermal shock experiments, Figure 9b shows that the Tg of the unfilled EP decreases rapidly. At the same time, the Tg of the TPU-filled composites remains higher than that of the unfilled epoxy.

These results suggest that TPU filling leads to a slight decrease in Tg for the EP composites. However, the Tg trend improves significantly with thermal shock for the TPU/EP composites. The unfilled EP exhibits the fastest Tg decrease, dropping by 12.2 °C. In contrast, the 10 wt% TPU composites show the smallest Tg reduction, with only a 0.2 °C decreases seen.

#### 3.3.3. Variable-Frequency Impedance Spectroscopy Test Results

**Figure 10:**
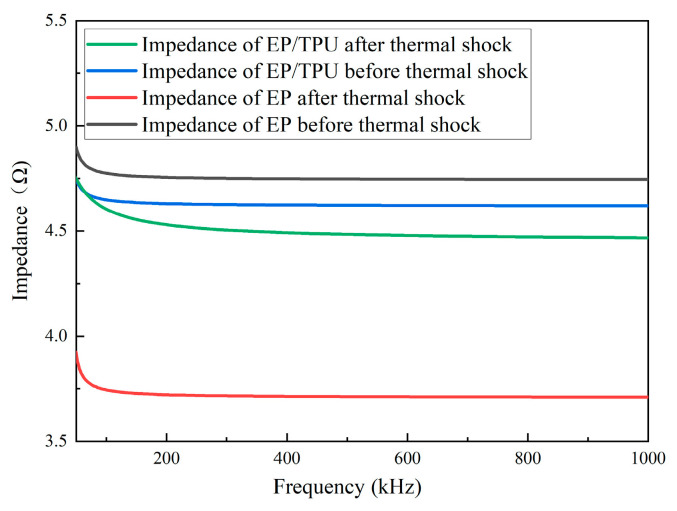
Results of variable-frequency impedance spectroscopy.

Variable-frequency impedance spectroscopy was performed on pure epoxy resin and the composite material containing 12.5 wt% filler before and after thermal shock. Figure 10 shows the trend in impedance variation with frequency for the four materials. As the frequency increases, the impedance gradually decreases for all materials. Before thermal shock, the impedance of the pure epoxy resin is higher than that of the filled composite material. However, after thermal shock, the impedance of the pure epoxy resin is lower than that of the filled composite material.

#### 3.3.4. Wideband Dielectric Spectroscopy Test Results

**Figure 11:**
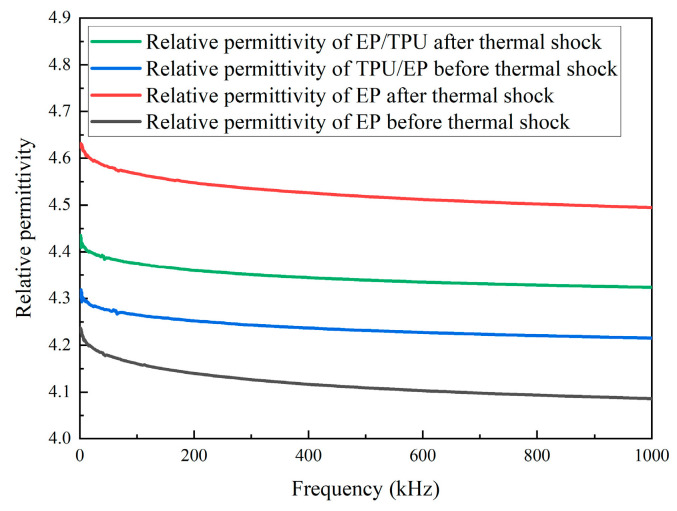
Results of broadband dielectric spectroscopy test.

Wideband dielectric spectroscopy was performed on pure epoxy resin and the composite material containing 12.5 wt% filler before and after thermal shock. Figure 11 shows the trend in dielectric constant variation with frequency for the four materials. As the frequency increases, the dielectric constant gradually decreases for all materials. Before thermal shock, the dielectric constant of the pure epoxy resin is higher than that of the filled composite material. However, after thermal shock, the dielectric constant of the pure epoxy resin is lower than that of the filled composite material. During the thermal shock process, the dielectric performance of the filled material is superior to that of pure epoxy resin.

#### 3.3.5. SEM Test Results

**Figure 12:**
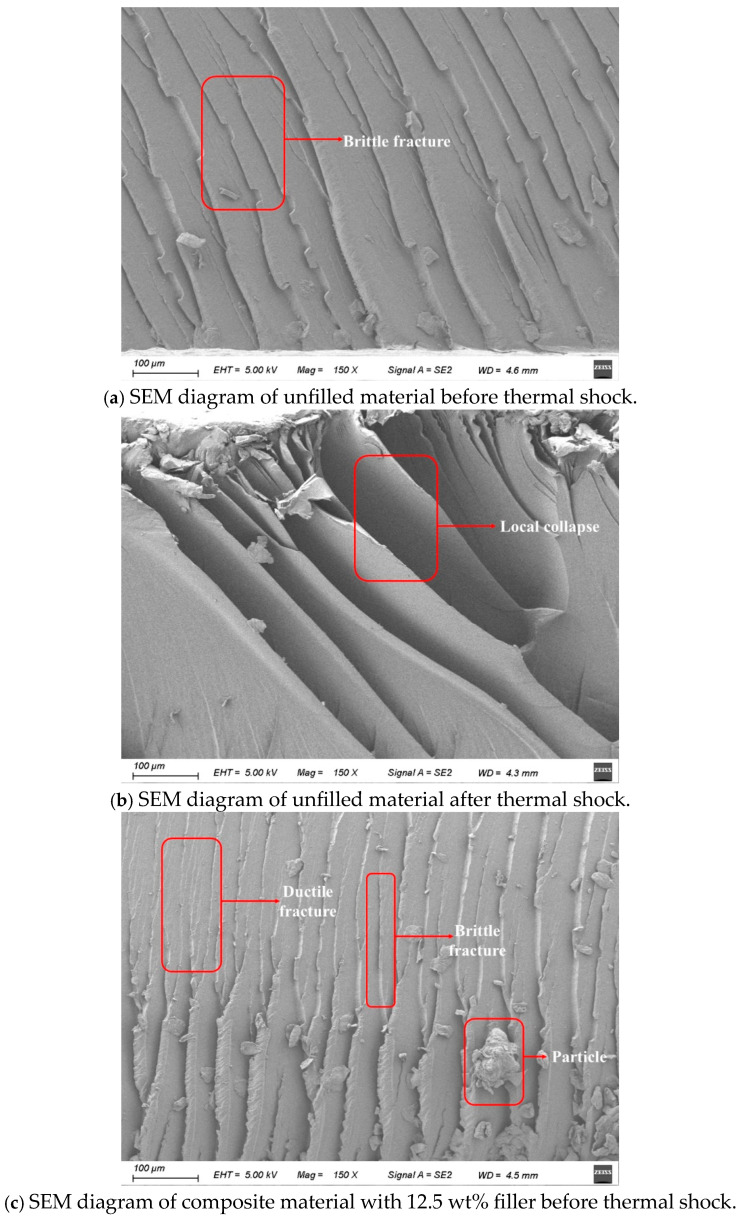

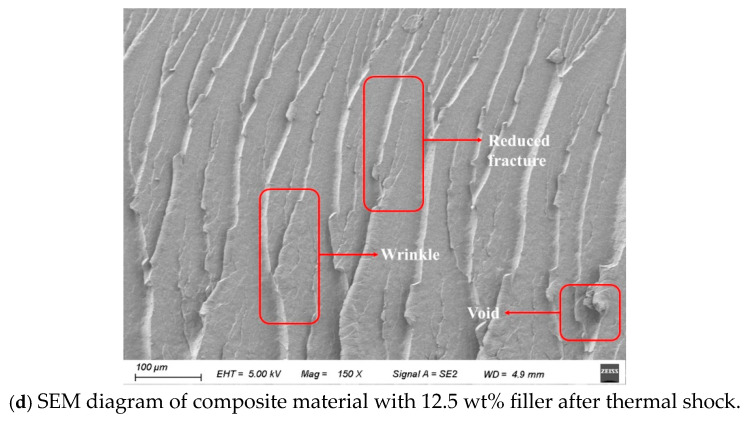
SEM diagrams of the composite material.

Samples with 0 wt% and 12.5 wt% filler contents were selected for the SEM analysis, as shown in Figure 10. Figure 12a depicts the fracture surface of the unfilled material before thermal shock, which displays brittle fracture characteristics. After thermal shock, Figure 12b shows that the unfilled material still exhibits brittle fracture, with larger cracks and some collapse. Figure 12c shows the 12.5 wt% filled sample before thermal shock; the fracture surface appears rough, with small particles present, indicating both brittle and ductile fracture. In Figure 12d, after thermal shock, the 12.5 wt% TPU-filled sample shows wrinkling and small pits, with reduced crack lengths and a smoother surface than before the shock. The filled material also shows fewer cracks than the unfilled one.

The results suggest that adding TPU changes the fracture mode from purely brittle to a mix of brittle and ductile. Thermal shock increases the cracks in the material, with unfilled samples showing more severe internal collapse while TPU-filled composites remain structurally intact without significant cracking or damage.

## 4. Analysis and Discussion

**Figure 13:**
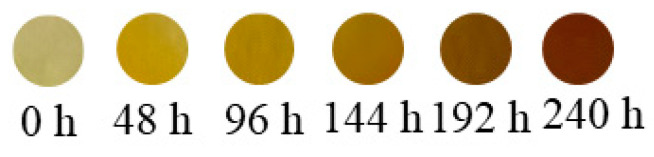
Samples before and after thermal shock.

Figure 13 shows the changes in the samples’ appearance after thermal shock. Their color gradually darkens, eventually turning amber. Fourier Transform Infrared (FTIR) analysis shows that the urethane thermally degrades into alkyl radicals. These radicals are further oxidized into aldehydes and carboxylic acids. The amines are oxidized into conjugated compounds, causing the samples to change color.

**Figure 14:**
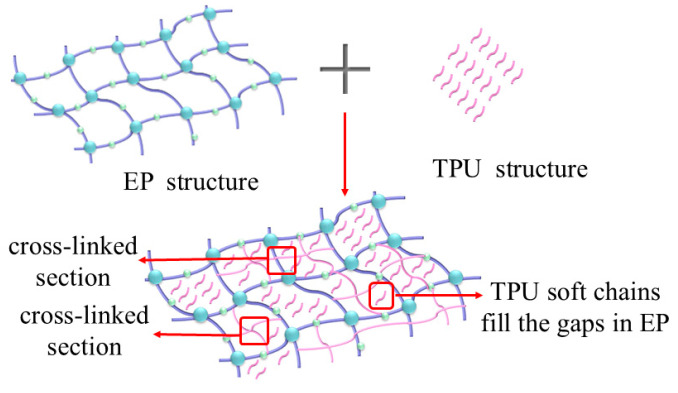
Process of reaction between TPU and EP.

As illustrated in Figure 14, during the stepwise curing process, the isocyanate groups (-NCO) in TPU exhibit high reactivity, readily engaging in nucleophilic addition reactions with hydroxyl groups (-OH) on the epoxy backbone. Additionally, the isocyanate groups (-N=C=O) undergo reaction with the epoxy groups (-C-O-C-), leading to the formation of urethane (HCOO-NH₂) and urea (-NH-CO-NH-) linkages. Urethane structures contribute to superior low-temperature flexibility, whereas urea moieties enhance thermal stability. The coexistence of these functional groups significantly enhances the mechanical integrity and thermal shock resistance of the composite material. Consequently, the TPU/EP composite matrix exhibits enhanced interfacial adhesion, ensuring the stability of its electrical properties.

EP has a high elastic modulus (approximately 1900 MPa) and a low elongation at break (around 4%). TPU has a low elastic modulus (about 200 MPa) and a high elongation at break (approximately 200%). Therefore, increasing the filler content can reduce the elastic modulus of the composite material and increase its elongation at break [6].

EP exhibits brittle fracture during tension, with its fracture surface appearing linear. Under an external impact, EP experiences brittle failure, damaging its fracture surface. TPU, as a flexible material, introduces flexible soft chains into the three-dimensional network structure of EP, forming a flexible composite [7]. Compared to traditional EP, TPU/EP composites show increased toughness. When subjected to external forces, the spherical or particle-like structures within the material help redistribute or disperse the applied stress. This action slows the propagation of cracks. Additionally, the flexible soft chains absorb the stress generated during crack propagation and distribute it to other areas, further slowing or preventing crack growth and material failure. A small amount of TPU can create a cross-linked structure within EP. The increased chemical cross-linking enhances the tensile and impact strength of the composite material [8]. However, excessive TPU addition may compromise the rigid support of EP’s molecular structure, leading to a decline in the composite’s mechanical properties.

Based on a microscopic morphology analysis, it was observed that after thermal shock, voids and cracks form on the surface and inside of the EP composite material. The fracture of the molecular chains in the material also generates a small amount of free radicals, increasing the number of charge carriers inside the material and leading to a decrease in its bulk resistivity. The small amount of TPU introduced into the EP matrix forms a cross-linking structure, which hinders ion transport and suppresses the decrease in the bulk resistivity of the composite material. According to the variable-frequency impedance spectroscopy analysis, after thermal shock, the epoxy resin material filled with TPU shows higher impedance compared to pure epoxy resin. TPU and epoxy resin have different dielectric constants and conductivity, which leads to charge accumulation at their interface, increasing the overall impedance of the composite, particularly in the low-frequency range. Thermal shock may exacerbate interface defects and interface polarization effects, causing the material to demonstrate a higher impedance in the variable-frequency test [9].

Moreover, thermal shock leads to the reorganization of charges within the composite material, resulting in a decrease in intermolecular forces, the breaking of some molecular chains, and the formation of polar groups and dipoles inside the material. Water molecules, introduced by cold shock, infiltrate the material, and their polarity induces dipole moments within the material [10], leading to increased polarization loss. The free radicals generated by molecular chain breakage will form vacancies and increase the number of free electrons inside the material, causing an increase in conductive losses. The impact also loosens the dielectric structure, which elevates structural losses. These phenomena result in an increase in both the relative dielectric constant and dielectric loss of the material. The essence of a dielectric response is a material’s ability to respond to an applied electric field through various types of polarization processes. However, different polarization mechanisms have distinct relaxation times, leading to varying contributions across different frequency ranges [11].

According to wideband dielectric spectroscopy, interface polarization dominates in the low-frequency region, leading to a higher dielectric constant. Due to the possible presence of free charge carriers inside the material, the dielectric loss is higher at low frequencies. In the mid-frequency range, dipolar polarization gradually becomes the dominant mechanism, but beyond their relaxation frequency, dipoles are unable to reorient promptly, resulting in a decrease in the dielectric constant. The polymer chains of TPU maintain better structural integrity after thermal shock, making the interface between the epoxy resin and filler more stable, reducing interface polarization [12] and improving dielectric stability. Additionally, the cross-linked structure formed between TPU and epoxy resin enhances intermolecular forces, reducing the molecular chain breakage caused by thermal shock, suppressing polarization loss and structural losses. A more compact structure also reduces the formation of material defects and vacancies after thermal shock, thus inhibiting charge movement and reducing conductive losses [13].

Additionally, the collapse of certain areas due to the leaching of small molecules after thermal shock can create micro-cracks [14]. This leads to molecular chain breakage, which facilitates electron flow. During cold shock, water molecules invade the material and form ice. This ice can further hydrolyze and create small molecular structures, promoting the formation of voids and cracks [15]. This process intensifies electron transfer and decreases the initiation voltage for partial discharge and the breakdown strength of the composite under short-duration AC conditions. Introducing TPU increases the material’s toughness and helps suppress crack formation and hydrolysis [16]. Its densely cross-linked structure further prevents a decline in electrical performance [17].

In summary, thermal shock can lower the glass transition temperature of EP materials, increase brittleness, and reduce impact resistance. This further leads to the formation of micro-cracks and larger cracks, which are the main reasons for the decline in the electrical performance of EP materials. TPU exhibits flexibility and elasticity [17], ensuring its mechanical properties remain effective in low-temperature environments. TPU also possesses excellent thermal stability, maintaining a stable electrical performance under high-temperature conditions. By introducing flexible TPU into the brittle EP [18], the brittleness of the EP can be reduced under thermal shock conditions. This improves the internal stress distribution and deformation of the material, enhancing its mechanical properties. Including TPU helps to distribute temperature changes more uniformly, reducing the likelihood of thermal stress concentrations in the EP composite and slowing the downward trend in the glass transition temperature. Enhancing toughness and stabilizing the glass transition temperature can prevent the material fracture and damage caused by thermal shock [18]. This also inhibits the formation of free electron pathways, improving the material’s dielectric properties [19]. Additionally, the tightly cross-linked structure formed between TPU and EP can mitigate the degradation of mechanical properties under thermal shock [20], reduce the leaching of small molecules, and slow down the decline in electrical insulation performance [21].

## 5. Conclusions

In the course of this study, EP/TPU composites were synthesized at varying concentrations and their mechanical and insulation properties were examined both before and after thermal shock. This investigation was further supported by microscopic analyses, leading to the following conclusions:

1. The incorporation of TPU into EP enhances the composite material’s mechanical properties. Under thermal shock conditions, compared to pure epoxy, the TPU/epoxy composite shows an increase in its elastic modulus of 10.1%, elongation at break of 15.3%, tensile strength of 22.3%, and impact strength of 47.8%.
2. Under thermal shock conditions, the TPU/EP composite exhibits an excellent electrical performance. With a 1–6.7 wt% TPU loading, materials subjected to 240 h of thermal cycling show a 10.5% increase in volume resistivity compared to pure epoxy, a 40.2% decrease in relative permittivity, an 85.5% reduction in dielectric loss, a 28.9% increase in partial discharge initiation voltage, and a 52.1% increase in AC breakdown strength.
3. The flexible molecular chains of TPU confer enhanced toughness and thermal stability to the material, enabling the TPU/EP composite to maintain a stable electrical performance during operation under fluctuating high and low temperatures. Furthermore, the cross-linked structure within the TPU/EP composite significantly mitigates the degradation of both mechanical and electrical properties when subjected to thermal shock conditions.

## Figures and Tables

**Table 1:** Raw materials and manufacturers.

Raw Material	Manufacturer
Bisphenol A EP E-51	Wuxi Boruiyu Chemical Technology
Thermoplastic Polyurethane Elastomer	BASF SE
N, N-DMF	Shanghai Pharmaceuticals
Methyl Tetahydrophthalic Anhydride	Guangzhou Zhonggao Chemical
Toughening Agent DH410	Jiaxing Dongfang Chemical Plant
Accelerator DMP-30	Jiaxing Dongfang Chemical Plant
Anhydrous Ethanol and Other Reagents	China National Pharmaceutical Group Chemical Reagents

## Data Availability

The original contributions presented in this study are included in the article. Further inquiries can be directed to the corresponding authors.
